# Knowledge, attitudes, and perception of the role of the media regarding COVID-19 in medical students from a peruvian university

**DOI:** 10.17843/rpmesp.2022.391.9702

**Published:** 2022-03-21

**Authors:** José Luis Paredes, Rafaella Navarro, Jorge Luis Andrade-Piedra, Noemí Hinostroza, Juan Echevarría, Camille Webb

**Affiliations:** 1 Instituto de Medicina Tropical Alexander von Humboldt, Universidad Peruana Cayetano Heredia, Lima, Peru. Universidad Peruana Cayetano Heredia Instituto de Medicina Tropical Alexander von Humboldt Universidad Peruana Cayetano Heredia Lima Peru; 2 Facultad de Medicina, Universidad Peruana Cayetano Heredia, Lima, Peru. Universidad Peruana Cayetano Heredia Facultad de Medicina Universidad Peruana Cayetano Heredia Lima Peru

**Keywords:** COVID-19, SARS-CoV-2, Knowledge, Medical Students, Peru, COVID-19 Serological Testing, Communications Media, Volunteers, Medicine

## Abstract

A cross-sectional study was carried out on medical students from a private Peruvian university. The aim was to describe knowledge and attitudes towards COVID-19 as well as the student’s perception of the role of media outlets and social media. Of the students, 32% did not know that during the first five days of illness, serological tests are preferred for diagnosing COVID-19 over molecular tests; 73% reported being willing to work as a volunteer during the pandemic, and 94% received false information regarding COVID-19 on social media. This study demonstrated that information regarding diagnostic tests should be reinforced and that the high percentage of students willing to volunteer during the COVID-19 pandemic should not be overlooked.

## INTRODUCTION

COVID-19 (Coronavirus Disease 2019) was declared a pandemic by the World Health Organization ^1^ ^-^ ^3^. The first case in Peru was reported on March 6, 2020 ^4^ and five days later the government implemented measures to control its spread. Classes in medical schools and hospital practices were suspended throughout Peru ^5^.

In the past, medical students have actively participated during outbreaks: during the Spanish flu pandemic they treated hundreds of patients; and during the polio epidemic they performed manual ventilation procedures ^6^. Despite the important role of medical students during health emergencies, they have shown little knowledge regarding disease transmission, symptomatology and diagnosis ^7^ ^,^ ^8^.

During the current COVID-19 pandemic, a systematic review on medical students, that included twenty studies, reported adequate knowledge about COVID-19 transmission, symptoms, and prevention. However, less than 50% of the students stated that the use of facemasks could decrease the transmission of COVID-19 and most of them stated that they seek information about COVID-19 on social networks ^9^.

In Peru, some studies have explored the knowledge of medical students about COVID-19, and found adequate knowledge about transmission, symptoms, and prevention measures ^10^. However, studies that address the attitudes of these students are needed to understand their willingness to volunteer during the pandemic, as well as their perception on biosafety training, and their perception of COVID-19 risk for health personnel, among other issues.

The aim of this study was to describe the level of knowledge and attitudes regarding COVID-19 of medical students from a private university in Lima during 2021; as well as their perception of the role of the media in informing about the disease.

**KEY MESSAGES**

Motivation for the study: To describe the level of knowledge, attitudes and perception of the role of the media regarding COVID-19 in medical students of a private university in Peru.

Main findings: We found an adequate level of knowledge about COVID-19; however, information about the use of diagnostic methods should be reinforced. Most were willing to volunteer during the COVID-19 pandemic and considered that the media are increasing fear, anxiety and confusion.

Public health implications: The high willingness of medical students to volunteer should be taken into account for future pandemic mitigation strategies.

## THE STUDY

A cross-sectional study was conducted in students from the first to the seventh year of medical school at the Universidad Peruana Cayetano Heredia, during 2021.

Non-probability convenience sampling was used. We calculated a sample size of 122 students considering a population of 1021 students using STATA v16.0, as well as 90% correct answers per question in the knowledge section ^10^, a confidence interval of 95%, and a precision of 5%. This sample size allowed us to determine the frequencies in the attitudes and perceptions sections with a precision of 8.9% considering a conservative frequency of 50% of correct answers.

The main variables in this study were the level of knowledge about COVID-19, attitudes towards COVID-19, and perception of the role of the media during the COVID-19 pandemic. In addition, we included sociodemographic variables.

Level of knowledge about COVID-19: the questions in this section were based on a study from Iran ^11^ and international guidelines ^1^ published up to August 2020. Initially, we considered 20 questions, then two questions were added from a study in Malaysia ^12^ and four from a study in the United States and the United Kingdom ^12^. Finally, the section consisted of 20 questions and the answer alternatives were: true, false and doesn’t know.

Attitudes about COVID-19: the questions in this section were based on a survey validated for MERS-CoV ^13^. It consisted of five questions with yes and no response options, and nine Likert-scale questions with response alternatives of: strongly agree, agree, neither agree nor disagree, disagree and strongly disagree. For better analysis, these five categories were grouped into three: disagree, indifferent and agree.

Perception of the role of the media: the questions in this section were adapted from a study on pharmacists in Jordan ^14^. It consisted of six Likert-scale questions with the following response alternatives: never, almost never, sometimes, almost always and always. For better analysis, these five categories were grouped into three: never/almost never, sometimes, almost always/always. Additionally, we included a question about false information on social networks, with yes and no response alternatives.

Sociodemographic variables: age, gender (male, female, non-binary), year of study (first or second, third fourth or fifth, sixth or seventh), living with older adults (<65 years) or people with comorbidities (obesity, arterial hypertension, diabetes and/or chronic kidney disease), current or previous history of COVID-19 and having taken a course on COVID-19.

A self-administered virtual survey (Annex 1) was used through the Google Forms platform between August and December 2020. The surveys were translated from English to Spanish independently by two translators, and these translations were reviewed by a third translator, who verified that they were correct. The survey was sent to all students through the university’s institutional mail, additionally it was disseminated through institutional social networks (Facebook and Instagram).

A review was carried out with a focus group of six medical students through Zoom (virtual meeting platform) to verify clarity and detect additional issues. The review was conducted by three infectious disease physicians, two general practitioners and two biologists who actively participated in the containment of the pandemic. The survey was sent to all students and they were asked for their opinions regarding the overall assessment, questions not understood, questions not needed, additional issues or questions to consider, and any additional comments. After the review, we determined that the students completed the survey in an average of 10 minutes. Additionally, one question was eliminated from the survey during the review.

The data from the surveys was exported to Excel. We used frequencies and percentages to summarize the categorical variables, and the median and interquartile range (IQR) for the numerical variables, due to their non-normal distribution. We used STATA version 16 for the statistical analysis.

The study was approved by the Ethics Committee of the Universidad Peruana Cayetano Heredia (approval code 456-26-20). Informed consent was included in the online form, prior to accessing the survey. No audio or video recording of the focus group was made during validation of the survey. Any identifiers were removed from the database prior to the analysis.

## RESULTS

A total of 107 students agreed to participate in the study. The median age was 20 years (IQR 19-21). Most were female (51.4%, n=55) and were taking basic science courses (first and second year) (50.5%, n=54). A total of 36 participants (35.6%) had previously taken a course on COVID-19 and four had COVID-19 previously (4.0%). Finally, 56 participants (52.3%) reported living with older adults or people with comorbidities (Table 1).

**Table 1:** Characteristics of the participants (n=107).

Characteristics	n (%)
Age, median (IQR)	20 (19-21)
Gender	
Male	51 (48.7)
Female	55 (51.4)
Non-binary	1 (0.9)
Year of study	
First or second	54 (50.5)
Third, fourth or fifth	39 (36.5)
Sixth or seventh	14 (13.0)
Received course on COVID-19	
Yes	36 (35.6)
No	71 (64.4)
Had COVID-19 previously	
Yes	4 (4.0)
No	97 (96.0)
Lives with older adults (<65 years) or people with comorbidities (obesity, high blood pressure, diabetes and/or chronic kidney disease).	
Yes	56 (52.3)
No	51 (47.7)

IQR: Interquartile range

Regarding knowledge about COVID-19, 38% (n=41) did not recognize that only during intubation, suction, bronchoscopy and cardiopulmonary resuscitation should healthcare personnel use the N95 ventilator, 31% (n=38) did not know that in the first 14 days of illness molecular tests are preferred over serological tests (Table 2).

**Table 2:** Level of knowledge about COVID-19 in medical students at a university in Lima, Peru (n=107).

N°	Questions (option considered as correct)	n (%)
1	COVID-19 is a respiratory infection caused by a new species of the coronavirus family. (True).	102 (95.3)
2	First case of COVID-19 was diagnosed in Wuhan, China (True).	106 (99.1)
3	The origin of COVID-19 is not yet fully defined, but it has been proposed that it comes from the consumption of seafood, snakes or bats (True).	91 (85.05)
4	The most common COVID-19 symptoms are fever, cough and shortness of breath. Other less common symptoms include nausea and diarrhea (True).	102 (95.3)
5	Incubation period is up to 14 days with an average of 5 days.	92 (85.9)
6	It can be diagnosed by PCR (Polymerase Chain Reaction) in samples collected from nasopharynx and oropharynx or from sputum or bronchial lavage (True).	94 (87.8)
7	Transmitted mainly by respiratory droplets when coughing or sneezing (True)	105 (98.1)
8	Transmitted by close contact with infected persons (e.g., at family gatherings, conglomerates, and healthcare facilities) (True).	104 (97.2)
9	This disease can be prevented by hand washing and proper personal hygiene (True)	100 (93.5)
10	The use of a surgical mask is useful to prevent the spread of the virus by coughing or sneezing (True)	103 (96.3)
11	Infection can be prevented by avoiding close contact such as shaking hands or kissing, by not attending meetings, and by constant hand disinfection (True).	107 (100)
12	Only during intubation, suction of secretions, bronchoscopy and cardiopulmonary resuscitation should healthcare personnel use the N95 respirator (True).	66 (61.7)
13	If a person has symptoms during the first 14 days after contact with a suspected case of COVID-19, he/she should go to a nearby health center (True).	69 (64.5)
14	People with COVID-19 do not infect others if they do not have a fever (False).	104 (97.2)
15	People infected with COVID-19, but who are asymptomatic, may also be contagious.	99 (92.5)
16	In the first 5 days of illness, serological tests (measuring antibodies) are better than molecular tests (polymerase chain reaction) for the diagnosis of COVID-19 (False).	69 (64.5)
17	Do you consider that people with comorbidities (other pre-existing conditions) have a higher risk of death from COVID-19 compared to people without comorbidities (True)?	105 (98.1)
18	Do you believe that only older adults can be infected by COVID-19? (False)	102 (95.3)
19	Due to COVID-19 infection, which age groups are most likely to die from the disease? (Adults over 60 years of age)	96 (89.7)
20	Herd immunity is a statistical phenomenon that occurs when a specific percentage of the population becomes immune to an infection, thus preventing an epidemic from growing. Do you consider that Peru has already reached herd immunity? (No)	83 (77.6)

Regarding attitudes, 78% (n=83) of the participants considered that healthcare personnel are being discriminated against because of their contact with patients with COVID-19. Also, 73% (n=78) would be willing to volunteer in order to help caring for patients during the pandemic (Table 3).

**Table 3:** Attitudes towards COVID-19 in medical students at a university in Lima, Peru (n=107).

N°	Questions	n (%)
1	Do you think that an effective treatment for COVID-19 will be found in the future?	
	Yes	93 (86.9)
	No	14 (13.1)
2	If you were infected with COVID-19, do you think it would be better to hide it from people to avoid discrimination?	
	Yes	8 (7.5)
	No	99 (92.5)
3	Do you consider that health personnel are currently being discriminated against because of their contact with patients with COVID-19?	
	Yes	83 (77.6)
	No	24 (22.4)
4	Would you be willing to care for patients infected with COVID-19 in the future?	
	Yes	104 (97.2)
	No	3 (2.8)
5	Would you be willing to volunteer to support patient care during the COVID-19 pandemic?	
	Yes	78 (72.9)
	No	29 (27.1)
6	Would you be afraid to work in places where patients with COVID-19 are admitted/treated?	
	Disagrees	39 (36.5)
	Neither agrees nor disagrees	39 (36.5)
	Agrees	29 (27.0)
7	Despite the use of personal protective equipment, the risk of COVID-19 is high in healthcare personnel	
	Disagrees	10 (9.3)
	Neither agrees nor disagrees	18 (16.8)
	Agrees	79 (73.9)
8	I believe that personal protective equipment and environments designated to care for patients with COVID-19 are not sufficient to prevent COVID-19 infection in healthcare personnel	
	Disagrees	19 (17.7)
	Neither agrees nor disagrees	17 (15.9)
	Agrees	71 (66.4)
9	I am afraid that a member of my family will contract COVID-19.	
	Disagrees	5 (4.6)
	Neither agrees nor disagrees	8 (7.5)
	Agrees	94 (87.9)
10	It is appropriate that schools and workplaces are closed because of COVID-19.	
	Disagrees	7 (6.5)
	Neither agrees nor disagrees	12 (11.2)
	Agrees	88 (82.3)
11	COVID-19 is highly transmissible in hospitals.	
	Disagrees	3 (2.8)
	Neither agrees nor disagrees	22 (20.6)
	Agrees	82 (76.6)
12	Health education has no effect on COVID-19 prevention.	
	Disagrees	100 (93.5)
	Neither agrees nor disagrees	4 (3.7)
	Agrees	3 (2.8)
13	I consider biosafety training regarding COVID-19 to be effective in protecting me in the event of exposure.	
	Disagrees	5 (4.7)
	Neither agrees nor disagrees	13 (12.1)
	Agrees	89 (83.2)
14	Caring for patients with COVID-19 is a threat to healthcare workers	
	Disagrees	21 (19.6)
	Neither agrees nor disagrees	47 (43.9)
	Agrees	39 (36.5)

Regarding medical students’ perception of the role of the media during the pandemic, 98% (n=101) reported receiving false information on social networks and more than 60% (n=68) strongly agreed/agreed that information provided by the media is adding to the fear, anxiety and confusion in the population (Table 4).

**Table 4:** Perceptions about the media of medical students at a university in Lima, Peru (n=103).

N°	Questions	n (%)
1	Do you consider that the media educate on procedures to follow during an epidemic?	
	Never/almost never	30 (29.1)
	Sometimes	36 (35.0)
	Almost always/always	37 (35.9)
2	Do you consider that the media disseminates preventive behaviors to help control COVID-19?	
	Never/almost never	16 (15.5)
	Sometimes	52 (50.5)
	Almost always/always	35 (34.0)
3	Do you consider that the media raise awareness about COVID-19?	
	Disagrees	32 (31.1)
	Sometimes	42 (40.8)
	Almost always/always	29 (28.1)
4	Do you consider that the media educates on how to care for a person with COVID-19?	
	Never/almost never	48 (46.6)
	Sometimes	42 (40.8)
	Almost always/always	13 (12.6)
5	Do you consider that the media is increasing fear, anxiety and confusion?	
	Never/almost never	13 (12.6)
	Sometimes	22 (21.4)
	Almost always/always	68 (66.0)
6	Do you trust the information in the media?	
	Never/almost never	47 (45.6)
	Sometimes	45 (43.7)
	Almost always/always	11 (10.7)
7	Have you received false information on social networks that could pose a health risk to our population regarding COVID-19 symptoms and treatment?	
	No	2 (2.0)
	Yes	101 (98.0)

## DISCUSSION

The study showed that students have adequate knowledge about COVID-19; however, there are important gaps in knowledge: one third of the students did not know that in the first 14 days of illness, molecular tests are preferred over serological tests. Most participants considered that healthcare personnel are being discriminated against because of their contact with COVID-19 patients, and one-third would not be willing to help in caring for patients during the pandemic.

Some studies from other settings ^10^ ^,^ ^15^ ^,^ ^16^ described that the knowledge of medical students regarding COVID-19 is adequate. In our study, 40% did not recognize that healthcare personnel should use the N95 ventilator only during intubation, suction, bronchoscopy and cardiopulmonary resuscitation; a study in Israel reported almost twice that percentage ^11^. We also found that the knowledge regarding diagnostic tests for COVID-19 is inadequate, which is similar to what was found by a study on Ecuadorian medical students (31% thought that the World Health Organization recommended using antibody tests). Both studies show the importance of informing students about COVID-19 diagnosis during the first seven days of the disease, which is when molecular tests (RT-PCR) are most sensitive.

Regarding attitudes, a high percentage were willing to care for patients with COVID-19 in the future (97%) as well as to volunteer to help in caring for patients during the pandemic (73%). Similar to our results, a study on medical interns in Peru found that almost 50% strongly agreed/agreed to return to the internship as volunteers if biosecurity measures were assured ^17^. Medical students worldwide are willing to volunteer during the current COVID-19 pandemic ^18^. In addition to providing clinical care, these students can assist in sample collection and processing as well as in dissemination of evidence on COVID-19 and contact tracing. Medical schools and the Peruvian Ministry of Health (MINSA) could build on these attitudes to strengthen the fight against COVID-19.

Most students reported receiving false information about COVID-19 on social media and perceived that the media adds to the fear, anxiety, and confusion produced by the disease. The dissemination of alarming and frightening messages to the population by fake news ^19^ ^) ^jeopardizes control of the pandemic. MINSA, in cooperation with media outlets, should carry out information campaigns to educate the population and to limit the dissemination of false news.

One of the limitations of our study is that the limited response of the students did not allow us to reach the expected sample size. Furthermore, selection bias is a possibility due to the use of convenience sampling, so the results should be carefully interpreted. Some recommendations may have changed since the beginning of the study up to the publication of this article. Because of these limitations, the results of our study may only be applied to the university where it was carried out, and could not be extrapolated to other contexts. We suggest that qualitative studies with larger samples should be carried out in order to delve deeper into these dimensions. Despite the limitations, our results are important and should be considered, such as those about the use of diagnostic tests and the possibility of volunteering.

In conclusion, although students have adequate knowledge regarding COVID-19, some topics should be reinforced. The high percentage of students willing to support in the fight against COVID-19 could be beneficial. These results could be used for the development of informative material on COVID-19 for medical students. Finally, we recommend the implementation of campaigns against misinformation and against the dissemination of false news in our population.

## References

[1] Centros para el Control y la Prevención de Enfermedades (2020). Aspectos básicos sobre el VIH y el COVID-19.

[2] Centers for Disease Control (2020). People with Certain Medical Conditions.

[3] Johns Hopkins University COVID-19 Dashboard by the Center for Systems Science and Engineering (CSSE) at Johns Hopkins University (JHU).

[4] Diario Oficial 'El Peruano' (2020). Decreto Supremo que declara Estado de Emergencia Nacional por las graves circunstancias que afectan la vida de la Nación a consecuencia del brote del COVID-19.

[5] Ministerio de Educación del Perú (2020). Comunicado - en atención al estado de emergencia en todo el país.

[6] Miller DG, Pierson L, Doernberg S (2020). The Role of Medical Students During the COVID-19 Pandemic. Ann Intern Med.

[7] Hisam A, Nadeem M, Mahmood-Ur-Rahman (2016). Knowledge and attitude regarding ebola virus disease among medical students of Rawalpindi a preventable threat not yet confronted. Pak J Med Sci.

[8] Tuohetamu S, Pang M, Nuer X, Mahemuti L, Mohemaiti P, Qin Y (2017). The knowledge, attitudes and practices on influenza among medical college students in Northwest China. Hum Vaccines Immunother.

[9] Magklara E, Angelis S, Solia E, Katsimantas A, Kourlaba G, Kostakis G (2021). The Role of Medical Students During COVID-19 Era A Review. Acta Bio Medica Atenei Parm.

[10] Paredes Pretell MJ (2020). Nivel de conocimientos sobre SARS CoV-2 en estudiantes de medicina humana de la Universidad Privada Antenor Orrego 2020.

[11] Taghrir MH, Borazjani R, Shiraly R (2020). COVID-19 and Iranian Medical Students; A Survey on Their Related-Knowledge, Preventive Behaviors and Risk Perception. Arch Iran Med.

[12] Azlan AA, Hamzah MR, Sern TJ, Hadi S, Mohamad E (2020). Public knowledge, attitudes and practices towards COVID-19 A cross-sectional study in Malaysia. PLoS One.

[13] Abdollahi M, Ghahramanian A, Shahbazi S, Rezaei F, Naghili Asghari-Jafarabadi M (2020). Developing a questionnaire to assess Iranian nurses' knowledge of and attitude to Middle East respiratory syndrome. East Mediterr Health J.

[14] Karasneh R, Al-Azzam S, Muflih S, Soudah O, Hawamdeh S, Khader Y (2021). Media's effect on shaping knowledge, awareness risk perceptions and communication practices of pandemic COVID-19 among pharmacists. Res Social Adm Pharm.

[15] Matthias AT, Padmasiri MSN, Dharani UGAN (2021). Knowledge, Attitudes, and Practices on COVID-19 Among Medical Students in Sri Lanka. Asia Pac J Public Health.

[16] Lincango-Naranjo E, Espinoza-Suarez N, Solis-Pazmino P, Vinueza-Moreano P, Rodriguez-Villafuerte S, Lincango-Naranjo J (2021). Paradigms about the COVID-19 pandemic knowledge, attitudes and practices from medical students. BMC Med Educ.

[17] Albitres-Flores L, Pisfil-Farroñay YA, Guillen-Macedo K, Niño-Garcia R, Alarcon-Ruiz A (2020). Percepción de los internos sobre la suspensión del internado médico durante la cuarentena por la COVID-19. Rev Peru Med Exp Salud Pública.

[18] Hagana A, Cecula P (2020). Medical Students in the Time of COVID-19 Opportunities and Challenges. AEM Educ Train.

[19] Orso D, Federici N, Copetti R, Vetrugno L, Bove T (2020). Infodemic and the spread of fake news in the COVID-19-era. Eur J Emerg Med.

