# Supplementary material for: Knowledge, attitudes, and perception of the role of the media regarding COVID-19 in medical students from a peruvian university
Source: Rev Peru Med Exp Salud Publica. 2022 Mar 21;39(1):70–6. doi: 10.17843/rpmesp.2022.391.9702 (PMC11397583; doi:10.17843/rpmesp.2022.391.9702)
Supplement: Supplementary material. — Available in the electronic version of the RPMESP. [file rpmesp-39-01-9702-s001.pdf]

MATERIAL SUPLEMENTARIO

ANEXO 1

Encuesta para estudiantes de Medicina sobre las características sociodemográficas, conocimientos, actitudes y percepción del rol de los medios de comunicación respecto al COVID-19.

Le pedimos que lea con atención las instrucciones de cada sección, se tome su tiempo para contestarla y marque siguiente para pasar de pregunta.

Datos generales

1. ¿Cuál es su edad? (escriba en números por favor)
2. ¿Cuál es su género? (marque con X su respuesta)
   Hombre □ Mujer □ Otro/no especifica □
3. ¿Cuál es su año de estudios? (marque con X su respuesta)
   Primero □ Segundo □Tercero □ Cuarto □ Quinto □ Sexto (externado)□
   Séptimo (internado) □
4. ¿Convive usted con adultos mayores o personas con comorbilidades que los pone en un mayor riesgo de una infección severa por COVID-19?
   Sí □ No □
5. ¿Ha tenido (o tiene actualmente) alguno de sus familiares COVID-19?
   Sí, un familiar tuvo COVID-19 en el pasado □ Sí, actualmente un familiar tiene COVID-19 □ No □
6. ¿Ha tenido (o tiene actualmente) usted COVID-19?
   Sí, tuve COVID-19 en el pasado □ Sí, actualmente tengo COVID-1 9□ No □

Conocimiento respecto al COVID-19

En la siguiente sección le realizaremos preguntas sobre su conocimiento respecto al COVID-19. Marque por favor verdadero (V), falso (F) o no sé (NS) en los siguientes enunciados según lo considere:

1. COVID-19 es una infección respiratoria causada por una especie nueva de la familia de los coronavirus.
   V □ F □ NS □

2. El primer caso de COVID-19 fue diagnosticado en Wuhan, China.
   V □ F □ NS □

3. El origen del COVID-19 aún no está esclarecido, pero se ha planteado que proviene del consumo de comida marina, serpientes o murciélagos.
   V □ F □ NS □

4. Los síntomas comunes del COVID-19 parecen ser fiebre, tos y falta de aire, síntomas poco frecuentes son náuseas y diarrea.
   V □ F □ NS □

5. El periodo de incubación es hasta 14 días con una media de cinco días.
   V □ F □ NS □

6. Se puede diagnosticar por PCR en muestras coleccionadas de nasofaringe y orofaringe o de esputo o lavado bronquial.
   V □ F □ NS □

7. Se transmite mediante gotitas respiratorias al toser o estornudar.
   V □ F □ NS □

8. Se transmite al estar en contacto cercano con personas infectadas (especialmente en familias, lugares conglomerados y centros de salud).
   V □ F □ NS □

9. Esta enfermedad puede ser prevenida al lavarse las manos y mantener una adecuada higiene personal.
   V □ F □ NS □

10. El uso de una mascarilla quirúrgica es útil para prevenir la propagación del virus al toser o estornudar
    V □ F □ NS □

11. La infección puede ser prevenida al evitar contacto cercano como el apretón de manos o los besos, al no ir a reuniones y mediante la frecuente desinfección de manos.
    V □ F □ NS □

12. Solo durante la entubación, succión de secreciones, broncoscopia y resucitación cardiopulmonar el personal de salud debería usar la mascarilla N95.
    V □ F □ NS □

13. La enfermedad puede ser tratada con fármacos antivirales.
    V □ F □ NS □

14. Si una persona tiene síntomas durante los primeros 14 días de haber tenido contacto con un caso sospechoso de COVID-19, debería acudir a un centro de salud cercano.
    V □ F □ NS □

15. Comparado con un resfrío común la congestión nasal y los estornudos son menos frecuentes en pacientes infectados por COVID-19.
V □ F □ NS □

16. Las personas con COVID-19 no infectan a los demás si no tienen fiebre.

V □ F □NS □

17. Las personas infectadas con COVID-19, pero que son asintomáticas, también pueden contagiar.

V □ F □ NS □

18. En los primeros cinco días de enfermedad, las pruebas serológicas (que miden anticuerpos) son mejores que las moleculares (reacción en cadena de polimerasa) para el diagnóstico de COVID-19.

V □ F □ NS □

19. ¿Considera usted que las personas con comorbilidades (otras enfermedades preexistentes) tienen un riesgo mayor de muerte por COVID-19 comparado con las personas sin comorbilidades?

V □ F □ NS □

20. ¿Considera usted que solo los adultos mayores pueden ser infectados por COVID-19?

V □ F □ NS □

21. La inmunidad de rebaño es un fenómeno estadístico que ocurre cuando un porcentaje específico de la población se vuelve inmune a una infección, haciendo que una epidemia ya no siga en crecimiento. ¿Considera usted que el Perú ya ha llegado a la inmunidad de rebaño?
V □ F □ NS □

22. Debido a la infección por COVID-19, ¿Qué grupos etarios tienen mayor probabilidad de morir debido a dicha enfermedad?
Jóvenes □ Adultos □ Adultos mayores de 60 años □ Todos □

Actitudes respecto al COVID-19

En la siguiente sección usted podrá contestar Sí, No o No sé, según corresponda.

1. ¿Considera usted que se encontrará un tratamiento efectivo contra el COVID-19 en el futuro?
   Sí □ No □ No sé □

2. Si estuviese usted infectado por COVID-19, ¿considera usted que sería mejor ocultárselo a las personas por miedo a la discriminación?
   Sí □ No □ No sé □

3. ¿Considera usted que el personal de salud esta siendo discriminado actualmente por su contacto con pacientes con COVID-19?
   Sí □ No □ No sé □

4. ¿Estaría usted dispuesto a atender a pacientes infectados con COVID-19 en el futuro?
   Sí □ No □ No sé □

5. ¿Estaría usted dispuesto a trabajar como voluntario para apoyar en la atención de pacientes durante la pandemia del COVID-19?
   Sí □ No □ No sé □

En las siguientes preguntas por favor marque usted del 1 al 5 siendo: 1 totalmente en desacuerdo, 2 en desacuerdo, 3 ni de acuerdo, ni en desacuerdo, 4 de acuerdo y 5 totalmente de acuerdo, en los siguientes enunciados.

6. Tendría miedo de trabajar en lugares donde pacientes con COVID-19 son admitidos/tratados.
   1 □ 2 □ 3 □ 4 □ 5 □

7. A pesar del uso de equipo de protección personal, el riesgo de contagio COVID-19 es alto en el personal de salud.
   1 □ 2 □ 3 □ 4 □ 5 □

8. Considero que los equipos de protección personal y los ambientes designados a atender a pacientes con COVID-19 no son suficientes para evitar la infección por COVID-19 en el personal de salud.
   1 □ 2 □ 3 □ 4 □ 5 □

9. Tengo miedo de que un miembro de mi familia contraiga COVID-19.
   1 □ 2 □ 3 □ 4 □ 5 □

10. En el caso de COVID-19 es adecuado que los colegios y centros de trabajo esten cerrados.
    1 □ 2 □ 3 □ 4 □ 5 □

11. El COVID-19 es sumamente transmisible en los hospitales.
    1 □ 2 □ 3 □ 4 □ 5 □

12. La educación en salud no tiene ningun efecto en la prevención de COVID-19.
    1 □ 2 □ 3 □ 4 □ 5 □

13. Considero que el entrenamiento que he recibido sobre COVID-19 es efectivo en protegerme de la enfermedad en caso de exposición.
    1 □ 2 □ 3 □ 4 □ 5 □

14. El atender a pacientes con COVID-19 podría ser una amenaza para el personal de salud.
    1 □ 2 □ 3 □ 4 □ 5□

Percepción de la calidad de información por los medios de comunicación

La siguiente sección trata sobre el rol de los medios de comunicación en la información de la pandemia por COVID-19. Por favor marque usted del 1 al 5 siendo: 1 nunca, 2 casi nunca, 3 a veces, 4 casi siempre y 5 siempre, en los siguientes enunciados. Finalmente, en la última pregunta marque usted Sí o No, según considere.

1. Los medios de comunicación educan a la población sobre los procedimientos a seguir en el contexto de un brote o una epidemia.
   1 □ 2 □ 3 □ 4 □ 5 □

2. Los medios de comunicación aumentan las conductas preventivas para controlar la infección.
   1 □ 2 □ 3 □ 4 □ 5 □

3. Los medios de comunicación aumentan el conocimiento sobre la COVID-19 en la comunidad.
   1 □ 2 □ 3 □ 4 □ 5 □

4. Los medios de comunicación educan sobre cómo proteger a otros si están enfermos o si tienen la sospecha de estar infectados por COVID-19.
   1 □ 2 □ 3 □ 4 □ 5 □

5. La información de los medios de comunicación aumenta el miedo, ansiedad y la confusión en la población.
   1 □ 2 □ 3 □ 4 □ 5 □

6. Usted confía en la información de los medios de comunicación.
   1 □ 2 □ 3 □ 4 □ 5 □

7. ¿Ha recibido usted información falsa en redes sociales que podría significar un riesgo para la salud de nuestra población respecto a síntomas y tratamiento de la COVID-19?
   Sí □ No□
