# Supplementary material for: Knowledge, attitudes, and perception of the role of the media regarding COVID-19 in medical students from a peruvian university
Source: Rev Peru Med Exp Salud Publica. 2022 Mar 21;39(1):70–6. doi: 10.17843/rpmesp.2022.391.9702 (PMC11397583; doi:10.17843/rpmesp.2022.391.9702)
Supplement: Translation

# Translation: Conocimientos, actitudes y percepción sobre el rol de los medios de comunicación respecto a la COVID-19 en estudiantes de Medicina de una universidad peruana

**Authors:** José Luis Paredes, Rafaella Navarro, Jorge Luis Andrade-Piedra, Noemí Hinostroza, Juan Echevarría, Camille Webb

**Affiliations:** 1 Instituto de Medicina Tropical Alexander von Humboldt, Universidad Peruana Cayetano Heredia, Lima, Perú.; 2 Facultad de Medicina, Universidad Peruana Cayetano Heredia, Lima, Perú.

**Keywords:** COVID-19, SARS-CoV-2, Conocimiento, Estudiantes de Medicina, Perú, Prueba Serológica para COVID-19, Medios de Comunicación, Voluntarios, Medicina

## Abstract

Se realizó un estudio transversal en estudiantes de Medicina de una universidad privada de Lima. El objetivo fue describir el nivel de conocimientos y las actitudes sobre la COVID-19, además de su percepción sobre el rol de los medios de comunicación y de las redes sociales. El 32% no sabía que en los primeros cinco días de la enfermedad, las pruebas serológicas son preferibles para diagnosticar la COVID-19, comparadas con las pruebas moleculares; el 73% reportó estar dispuesto a trabajar como voluntario durante la pandemia y el 94% recibió información falsa sobre la COVID-19 en las redes sociales. Este estudio demuestra que la información sobre el uso de pruebas diagnósticas debe ser reforzada y que se debe tomar en cuenta el alto porcentaje de estudiantes dispuestos a ser voluntarios durante la pandemia de la COVID-19.

## INTRODUCCIÓN

La COVID-19 (Coronavirus Disease 2019) fue declarada pandemia por la Organización Mundial de la Salud ^1^ ^-^ ^3^. El primer caso en el Perú fue anunciado el 6 de marzo de 2020 ^4^ y cinco días después se implementaron medidas para controlar su expansión. Las clases presenciales en las facultades de Medicina y las prácticas hospitalarias se suspendieron en todo el Perú ^5^. 

En el pasado los estudiantes de Medicina han participado activamente durante epidemias: en la gripe española atendieron a cientos de pacientes y en la epidemia de polio realizaron procedimientos de ventilación manual ^6^. A pesar del importante rol de los estudiantes de Medicina en las emergencias sanitarias, han demostrado tener poco conocimiento sobre la transmisión, sintomatología y diagnóstico de las enfermedades ^7^ ^,^ ^8^.

En la actual pandemia de la COVID-19, una revisión sistemática que incluyó veinte estudios reportó un adecuado conocimiento sobre la transmisión, los síntomas y la prevención de la COVID-19 en estudiantes de Medicina. Sin embargo, menos de 50% de los estudiantes afirmó que el uso de mascarillas podía disminuir la transmisión de la COVID-19 y la mayoría de ellos declaró que busca información sobre COVID-19 en redes sociales ^9^. 

En Perú, algunos estudios han explorado el conocimiento de estudiantes de Medicina sobre la COVID-19, y encontraron un adecuado conocimiento sobre la transmisión, los síntomas y las medidas de prevención ^10^. Sin embargo, se necesitan estudios que aborden las actitudes de estos estudiantes para entender su predisposición de trabajar como voluntarios durante la pandemia, la percepción del entrenamiento en bioseguridad y la percepción del riesgo de COVID-19 para personal de salud, entre otros temas. 

El objetivo de este estudio fue describir el nivel de conocimientos y las actitudes respecto a la COVID-19; así como su percepción sobre el rol de los medios de comunicación en la información sobre la enfermedad en estudiantes de Medicina de una universidad privada en Lima durante el 2021.

**MENSAJES CLAVE**

Motivación para realizar el estudio: describir el nivel de conocimientos, actitudes y la percepción del rol de los medios de comunicación respecto a la COVID-19 en estudiantes de medicina de una universidad privada del Perú.

Principales hallazgos: se encontró un buen nivel de conocimiento sobre la COVID-19; sin embargo, la información sobre el uso de métodos diagnósticos debe ser reforzada. La mayoría estuvo dispuesto a ser voluntario durante la pandemia de la COVID-19 y consideraron que los medios de comunicación están aumentando el miedo, la ansiedad y la confusión.

Implicancias en salud pública: la alta disponibilidad de los estudiantes de Medicina para ser voluntarios debe tomarse en cuenta para futuras estrategias de mitigación de la pandemia.

## EL ESTUDIO

Se realizó un estudio transversal en alumnos del primer al séptimo año de la carrera de Medicina de la Universidad Peruana Cayetano Heredia, durante el 2021.

Se utilizó un muestreo no probabilístico por conveniencia. Se calculó un tamaño de muestra de 122 estudiantes considerando una población de 1021 estudiantes usando STATA v16.0, 90% de respuestas acertadas por pregunta en la sección de conocimientos ^10^, un intervalo de confianza de 95%, y una precisión del 5%. Este tamaño muestral permitió determinar las frecuencias en las secciones de actitudes y percepciones con una precisión del 8,9% considerando una frecuencia conservadora del 50% de respuestas acertadas.

Las variables principales de este estudio fueron el nivel de conocimientos sobre la COVID-19, las actitudes frente a la COVID-19 y la percepción del rol de los medios de comunicación durante la pandemia de COVID-19. Además, se incluyeron variables sociodemográficas.

Nivel de conocimientos sobre la COVID-19: las preguntas de esta sección estuvieron basadas en un estudio de Irán ^11^ y de guías internacionales ^1^ publicadas hasta agosto de 2020. Al principio se consideraron 20 preguntas, luego se añadieron dos preguntas de un estudio en Malasia ^12^ ^) ^y cuatro de un estudio de Estados Unidos y de Reino Unido ^12^. Finalmente, quedo conformada por 20 preguntas con opciones de respuesta de verdadero, falso y no sé.

Actitudes sobre la COVID-19: las preguntas de esta sección estuvieron basadas en una encuesta validada para el MERS-CoV ^13^. Quedo conformada por cinco preguntas con opciones de respuesta de sí y no, y nueve preguntas en escala Likert con alternativas de respuesta de: totalmente de acuerdo, de acuerdo, ni en acuerdo ni en desacuerdo, en desacuerdo y totalmente en desacuerdo. Para un mejor análisis, estas cinco categorías se agruparon en tres: en desacuerdo, indiferente y de acuerdo.

Percepción del rol de los medios de comunicación: las preguntas de esta sección fueron adaptadas de un estudio en farmacéuticos de Jordania ^14^. Constó de seis preguntas en escala Likert con alternativas de respuesta de: nunca, casi nunca, a veces, casi siempre y siempre. Para un mejor análisis, estas cinco categorías se agruparon en tres: nunca/casi nunca, a veces, casi siempre/siempre. Adicionalmente, se incluyó una pregunta sobre información falsa en las redes sociales, con alternativas de respuesta de sí y no.

Variables sociodemográficas: edad, género (masculino, femenino, no binario), año de estudio (primero o segundo, tercero cuarto o quinto, sexto o séptimo), convive con adultos mayores (<65 años) o con personas con comorbilidades (obesidad, hipertensión arterial, diabetes y/o enfermedad renal crónica), haber tenido o tener COVID-19 y haber llevado un curso sobre COVID-19.

Se utilizó una encuesta virtual autoadministrada (Anexo 1) a través de la plataforma Google Forms entre agosto y diciembre 2020. Las encuestas fueron traducidas del inglés al español de forma independiente por dos traductores, y estas traducciones fueron revisadas por un tercer traductor, quien verificó que sean correctas. La encuesta fue enviada a todos los estudiantes a través del correo institucional de la universidad, adicionalmente se difundió a través de redes sociales institucionales (Facebook e Instagram). 

Se realizó la revisión con un grupo focal de seis estudiantes de Medicina a través de Zoom (plataforma de reuniones virtuales), para verificar la claridad y detectar temas adicionales. La revisión fue realizada por tres médicos infectólogos, dos médicos generales y dos biólogos que participaron activamente en la contención de la pandemia. A todos se les envió la encuesta y se consultaron sus opiniones respecto a la valoración general, preguntas que no se entiendan, preguntas no necesarias, temas o preguntas adicionales que considerar, y algún comentario adicional. Después de la revisión se determinó el tiempo promedio de diez minutos para la realización de la encuesta. Adicionalmente, en la revisión se eliminó una pregunta de la encuesta.

Se exportó la información de las encuestas a Excel. Se utilizaron frecuencias y porcentajes para resumir las variables categóricas y la mediana y el rango intercuartílico (RIC) para las variables numéricas, por su distribución no normal. Para el análisis estadístico se utilizó el programa STATA version16.

El estudio fue aprobado por el Comité de Ética de la Universidad Peruana Cayetano Heredia (código de aprobación 456-26-20). En el formulario virtual se incorporó el consentimiento informado, previo al acceso a la encuesta. No se realizó la grabación de audio o video del grupo focal durante la validación de la encuesta. Antes del análisis se eliminó cualquier identificador de la base de datos. 

## RESULTADOS

Un total de 107 estudiantes aceptaron participar del estudio. La mediana de edad fue de 20 años (RIC 19-21). La mayoría fueron mujeres (51,4%, n=55) y cursaban ciencias básicas (primer y segundo año) (50,5%, n=54). Un total de 36 participantes (35,6%) habían llevado un curso sobre COVID-19 y cuatro enfermaron con la COVID-19 en el pasado (4,0%). Finalmente, 56 participantes (52,3%) reportaron convivir con adultos mayores o con personas con comorbilidades (Tabla 1).

**Tabla 1:** Características de los participantes (n=107).

Características	n (%)
Edad, mediana (RIC)	20 (19-21)
Género	
Masculino	51 (48,7)
Femenino	55 (51,4)
No-binario	1 (0,9)
Año de estudios	
Primer o segundo	54 (50,5)
Tercer, cuarto o quinto	39 (36,5)
Sexto o séptimo	14 (13,0)
Recibió curso sobre la COVID-19	
Sí	36 (35,6)
No	71 (64.4)
Tuvo COVID-19 en el pasado	
Sí	4 (4,0)
No	97 (96,0)
Convive con adultos mayores (<65 años) o con personas con comorbilidades (obesidad, hipertensión arterial, diabetes y/o enfermedad renal crónica)	
Sí	56 (52,3)
No	51 (47,7)

RIC= rango intercuartílico

Con respecto a los conocimientos sobre la COVID-19, el 38% (n=41) no reconoció que solo durante la intubación, succión de secreciones, broncoscopia y resucitación cardiopulmonar el personal de salud debería usar el respirador N95 y el 31% (n=38) no sabía que en los primeros 14 días de enfermedad las pruebas serológicas no son preferibles en comparación con las moleculares (Tabla 2). 

**Tabla 2:** Nivel de conocimientos sobre la COVID-19 en estudiantes de Medicina de una universidad en Lima, Perú (n=107).

N°	Preguntas (opción considerada como correcta)	n (%)
1	El COVID-19 es una infección respiratoria causada por una especie nueva de la familia de los coronavirus. (Verdadero)	102 (95,3)
2	El primer caso de COVID-19 fue diagnosticado en Wuhan, China (Verdadero)	106 (99,1)
3	El origen de la COVID-19 aún no está completamente definido, pero se ha planteado que proviene del consumo de comida marina, serpientes o murciélagos (Verdadero)	91 (85,05)
4	Los síntomas más comunes del COVID-19 son fiebre, tos y falta de aire. Otros síntomas menos frecuentes incluyen náuseas y diarrea. (Verdadero)	102 (95,3)
5	El periodo de incubación es hasta 14 días con una media de 5 días	92 (85,9)
6	Se puede diagnosticar por PCR (Reacción en cadena de la polimerasa) en muestras coleccionadas de nasofaringe y orofaringe o de esputo o lavado bronquial (Verdadero)	94 (87,8)
7	Se transmite principalmente mediante gotitas respiratorias al toser o estornudar (Verdadero)	105 (98,1)
8	Se transmite al estar en contacto cercano con personas infectadas (por ejemplo, en reuniones familiares, lugares conglomerados y centros de salud) (Verdadero)	104 (97,2)
9	Esta enfermedad puede ser prevenida al lavarse las manos y mantener una adecuada higiene personal (Verdadero)	100 (93,5)
10	El uso de una mascarilla quirúrgica es útil para prevenir la propagación del virus al toser o estornudar (Verdadero)	103 (96,3)
11	La infección puede ser prevenida al evitar contacto cercano como el apretón de manos o los besos, al no ir a reuniones y mediante la frecuente desinfección de manos (Verdadero)	107 (100)
12	Solo durante la intubación, succión de secreciones, broncoscopia y resucitación cardiopulmonar el personal de salud debería usar el respirador N95 (Verdadero)	66 (61,7)
13	Si una persona tiene síntomas durante los primeros 14 días de haber tenido contacto con un caso sospechoso de COVID-19, debería acudir a un centro de salud cercano (Verdadero)	69 (64,5)
14	Las personas con COVID-19 no infectan a los demás si no tienen fiebre (Falso)	104 (97,2)
15	Las personas infectadas con COVID-19, pero que son asintomáticas, también pueden contagiar	99 (92,5)
16	En los primeros 5 días de enfermedad, las pruebas serológicas (que miden anticuerpos) son mejores que las moleculares (reacción en cadena de polimerasa) para el diagnóstico de COVID-19 (Falso)	69 (64,5)
17	¿Considera usted que las personas con comorbilidades (otras enfermedades preexistentes) tienen un riesgo mayor de muerte por COVID-19 comparado con las personas sin comorbilidades? (Verdadero)	105 (98,1)
18	¿Considera usted que solo los adultos mayores pueden ser infectados por COVID-19? (Falso)	102 (95,3)
19	Debido a la infección por COVID-19, ¿Qué grupos etarios tienen mayor probabilidad de morir debido a dicha enfermedad? (Adultos mayores de 60 años)	96 (89,7)
20	La inmunidad de rebaño es un fenómeno estadístico que ocurre cuando un porcentaje específico de la población se vuelve inmune a una infección, haciendo que una epidemia ya no siga en crecimiento. ¿Considera usted que el Perú ya ha llegado a la inmunidad de rebaño? (No)	83 (77,6)

Respecto a las actitudes, el 78% (n=83) de los participantes consideraba que el personal de salud está siendo discriminado por su contacto con pacientes con la COVID-19. Asimismo, el 73% (n=78) estaría dispuesto a trabajar como voluntario para apoyar en la atención de pacientes durante la pandemia (Tabla 3).

**Tabla 3:** Actitudes frente a la COVID-19 en estudiantes de Medicina de una universidad de Lima, Perú (n=107).

N°	Preguntas	n (%)
1	¿Considera usted que se encontrará un tratamiento efectivo contra el COVID-19 en el futuro?	
	Sí	93 (86,9)
	No	14 (13,1)
2	Si estuviese infectado por COVID-19, ¿Considera usted que sería mejor ocultárselo a las personas para evitar discriminación?	
	Sí	8 (7,5)
	No	99 (92,5)
3	¿Considera usted que el personal de salud está siendo discriminado actualmente por su contacto con pacientes con COVID-19?	
	Sí	83 (77,6)
	No	24 (22,4)
4	¿Estaría usted dispuesto o dispuesta a atender a pacientes infectado con COVID-19 en el futuro?	
	Sí	104 (97,2)
	No	3 (2,8)
5	¿Estaría usted dispuesto o dispuesta a trabajar como voluntario para apoyar en la atención de pacientes durante la pandemia de COVID-19?	
	Sí	78 (72,9)
	No	29 (27,1)
6	Tendría miedo de trabajar en lugares donde pacientes con COVID-19 son admitidos/tratados	
	En desacuerdo	39 (36,5)
	Indiferente	39 (36,5)
	De acuerdo	29 (27,0)
7	A pesar del uso de equipo de protección personal, el riesgo de COVID-19 es alto en personal de salud	
	En desacuerdo	10 (9,3)
	Indiferente	18 (16,8)
	De acuerdo	79 (73,9)
8	Considero que los equipos de protección personal y los ambientes designados para atender a pacientes con COVID-19 no son suficientes para evitar la infección por COVID-19 en el personal de salud	
	En desacuerdo	19 (17,7)
	Indiferente	17 (15,9)
	De acuerdo	71 (66,4)
9	Tengo miedo de que un miembro de mi familia contraiga COVID-19	
	En desacuerdo	5 (4,6)
	Indiferente	8 (7,5)
	De acuerdo	94 (87,9)
10	En el caso del COVID-19 es adecuado que los colegios y centro de trabajo estén cerrados	
	En desacuerdo	7 (6,5)
	Indiferente	12 (11,2)
	De acuerdo	88 (82,3)
11	El COVID-19 es sumamente transmisible en los hospitales	
	En desacuerdo	3 (2,8)
	Indiferente	22 (20,6)
	De acuerdo	82 (76,6)
12	La educación en salud no tiene ningún efecto en la prevención del COVID-19	
	En desacuerdo	100 (93,5)
	Indiferente	4 (3,7)
	De acuerdo	3 (2,8)
13	Considero que el entrenamiento en bioseguridad respecto al COVID-19 es efectivo para protegerme de la enfermedad en caso de exposición	
	En desacuerdo	5 (4,7)
	Indiferente	13 (12,1)
	De acuerdo	89 (83,2)
14	El atender a pacientes con COVID-19 es una amenaza para el personal de salud	
	En desacuerdo	21 (19,6)
	Indiferente	47 (43,9)
	De acuerdo	39 (36,5)

Respecto a la percepción de los estudiantes de Medicina sobre el rol de los medios de comunicación durante la pandemia, el 98% (n=101) reportó haber recibido información falsa en redes sociales y más del 60% (n=68) estuvieron totalmente de acuerdo/de acuerdo en que la información de los medios de comunicación está aumentando el miedo, ansiedad y la confusión en la población (Tabla 4). 

**Tabla 4:** Percepciones sobre los medios de comunicación de estudiantes de Medicina de una universidad de Lima, Perú (n=103).

N°	Preguntas	n (%)
1	¿Considera usted que los medios de comunicación educan sobre procedimientos a seguir durante una epidemia?	
	Nunca/casi nunca	30 (29,1)
	A veces	36 (35,0)
	Casi siempre/siempre	37 (35,9)
2	¿Considera usted que los medios de comunicación difunden conductas preventivas para controlar el COVID-19?	
	Nunca/casi nunca	16 (15,5)
	A veces	52 (50,5)
	Casi siempre/siempre	35 (34,0)
3	¿Considera usted que los medios de comunicación aumentan el conocimiento sobre COVID-19?	
	En desacuerdo	32 (31,1)
	A veces	42 (40,8)
	Casi siempre/siempre	29 (28,1)
4	¿Considera usted que los medios de comunicación educan sobre cómo cuidar a una persona con COVID-19?	
	Nunca/casi nunca	48 (46,6)
	A veces	42 (40,8)
	Casi siempre/siempre	13 (12,6)
5	¿Considera usted que los medios de comunicación están aumentando el miedo, la ansiedad y la confusión?	
	Nunca/casi nunca	13 (12,6)
	A veces	22 (21,4)
	Casi siempre/siempre	68 (66,0)
6	¿Confía usted en la información de los medios de comunicación?	
	Nunca/casi nunca	47 (45,6)
	A veces	45 (43,7)
	Casi siempre/siempre	11 (10,7)
7	¿Ha recibido usted información falsa en redes sociales que podría significar un riesgo para la salud de nuestra población respecto a síntomas y tratamiento del COVID-19?	
	No	2 (2,0)
	Sí	101 (98,0)

## DISCUSIÓN

Este estudio demostró un buen conocimiento sobre la COVID-19 en estudiantes de Medicina; sin embargo, existen brechas importantes en el conocimiento: un tercio de los estudiantes no sabía que en los primeros 14 días de enfermedad las pruebas serológicas no son preferibles en comparación a las moleculares. La mayoría consideró que el personal de salud está siendo discriminado por su contacto con pacientes con la COVID-19, y un tercio no estaría dispuesto a trabajar como voluntario para apoyar en la atención de pacientes durante la pandemia. 

Algunos estudios de otros contextos ^10^ ^,^ ^15^ ^,^ ^16^ describieron un adecuado conocimiento sobre la COVID-19 en estudiantes de Medicina. En este estudio, el 40% no reconoció que solo durante la intubación, succión de secreciones, broncoscopia y resucitación cardiopulmonar el personal de salud debería usar el respirador N95, casi el doble del porcentaje reportado en un estudio en Israel ^11^. Asimismo, el bajo conocimiento sobre pruebas diagnósticas para COVID-19 en este estudio es similar a los hallazgos en estudiantes de Medicina ecuatorianos (31% pensaba que la Organización Mundial de la Salud recomendaba usar pruebas de anticuerpos). Ambos estudios denotan la importancia de informar a los estudiantes sobre el diagnóstico de COVID-19 durante los primeros siete días de enfermedad, tiempo en el que las pruebas moleculares (RT-PCR) son más sensibles. 

Respecto a las actitudes, un alto porcentaje estaba dispuesto a atender a pacientes con la COVID-19 en el futuro (97%) y a trabajar como voluntario para apoyar en la atención de pacientes durante la pandemia (73%). Estos hallazgos son similares a un estudio en internos de Medicina en Perú, en el que casi el 50% estaba totalmente de acuerdo/de acuerdo en retornar al internado como voluntario si se aseguraban las medidas de bioseguridad ^17^. En todo el mundo los estudiantes de Medicina están dispuestos a apoyar como voluntarios durante la actual pandemia de la COVID-19 ^18^. Además de brindar atención clínica, estos estudiantes pueden apoyar en la colección y procesamiento de muestras, la diseminación de la evidencia sobre la COVID-19 y el seguimiento de contactos. Las escuelas de Medicina y el Ministerio de Salud del Perú (MINSA) podrían aprovechar estas actitudes para reforzar la lucha contra la COVID-19. 

La mayoría de los estudiantes reportó haber recibido información falsa sobre la COVID-19 en redes sociales y percibía que los medios de comunicación estaban aumentando el miedo, la ansiedad y la confusión respecto a esta enfermedad. Las noticias falsas han ocasionado la difusión de mensajes alarmantes y aterradores a la población ^19^, y se está poniendo en peligro el control de la pandemia. Es fundamental que el MINSA, en compañía de los medios de comunicación, realicen campañas informativas para educar a la población y para limitar la diseminación de noticias falsas. 

Se deben mencionar algunas limitaciones de este estudio: No se alcanzó el tamaño de muestra por la limitada respuesta de los estudiantes. La posibilidad de sesgo de selección al haber utilizado un muestreo por conveniencia, por lo que estos hallazgos deben ser interpretados con cautela. Algunas recomendaciones pueden haber cambiado desde el inicio del estudio hasta la fecha de publicación del artículo. Debido a estas limitaciones, los resultados solo serian aplicables a la universidad donde se realizó el estudio y no serían extrapolables a otras realidades. Consideramos que se deben realizar estudios con un mayor tamaño de muestra y con diseños cualitativos para poder ahondar en estas dimensiones. A pesar de las limitaciones, el estudio muestra resultados importantes que deben ser considerados, como el uso de pruebas diagnósticas y las posibilidades de voluntariado. 

En conclusión, a pesar de que los estudiantes conocen la mayoría de los aspectos sobre la COVID-19, algunos temas deben ser reforzados. El alto porcentaje de estudiantes dispuestos a apoyar en la lucha contra la COVID-19 debería ser aprovechado. Estos resultados podrían servir para el desarrollo de material informativo sobre la COVID-19 para estudiantes de Medicina. Finalmente, recomendamos la realización de campañas contra la desinformación y contra la difusión de noticias falsas en nuestra población.
